# Relationship between facility number of clinicians and prescribing intensity of psychotropic medications, opioids, and antibiotics in ambulatory practice

**DOI:** 10.1186/s12913-024-10613-z

**Published:** 2024-02-16

**Authors:** Hyungjin Myra Kim, Julie Strominger, Kara Zivin, Tony Van, Donovan T. Maust

**Affiliations:** 1 Consulting for Statistics, Computing and Analytics Research, University of Michigan, 915 E. Washington Street, Ann Arbor, Michigan 48109-1070 USA; 2 Center for Clinical Management Research, VA Ann Arbor Health System, Ann Arbor, Michigan 48105 USA; 3 Department of Psychiatry, Michigan Medicine, Ann Arbor, Michigan 40109 USA

**Keywords:** Prescribing intensity, Number of clinicians, Psychotropics, Antibiotics, Opioids

## Abstract

**Background:**

Promoting appropriate pharmacotherapy requires understanding the factors that influence how clinicians prescribe medications. While prior work has focused on patient and clinician factors, features of the organizational setting have received less attention, though identifying sources of variation in prescribing may help identify opportunities to improve patient safety and outcomes.

**Objective:**

To evaluate the relationship between the number of clinicians who prescribe medications in a facility and facility prescribing intensity of six individual medication classes by clinician specialty: benzodiazepines, antipsychotics, antiepileptics, and antidepressants by psychiatrists and antibiotics, opioids, antiepileptics, and antidepressants by primary care clinicians (PCPs).

**Design:**

We used 2017 Veterans Health Administration (VHA) administrative data.

**Subjects:**

We included patient-clinician dyads of older patients (> 55 years) with an outpatient encounter with a clinician in 2017. Patient-clinician data from 140 VHA facilities were included (*n* = 13,347,658). Analysis was repeated for years 2014 to 2016.

**Main measures:**

For each medication, facility prescribing intensity measures were calculated as clinician prescribing intensity averaged over all clinicians at each facility. Clinician prescribing intensity measures included percentage of each clinician’s patients prescribed the medication and mean number of days supply per patient among all patients of each clinician.

**Key results:**

As the number of prescribing clinicians in a facility increased, the intensity of prescribing decreased. Every increase of 10 facility clinicians was associated with a significant decline in prescribing intensity for both specialties for different medication classes: for psychiatrists, declines ranged from 6 to 11%, and for PCPs, from 2 to 3%. The pattern of more clinicians less prescribing was significant across all years.

**Conclusion:**

Future work should explore the mechanisms that link the number of facility clinicians with prescribing intensity for benzodiazepines, antipsychotics, antiepileptics, antidepressants, antibiotics, and opioids. Facilities with fewer clinicians may need additional resources to avoid unwanted prescribing of potentially harmful or unnecessary medications.

**Supplementary Information:**

The online version contains supplementary material available at 10.1186/s12913-024-10613-z.

To understand and improve pharmacotherapy, prior work has used the concept of intensity to capture the likelihood of a particular medication or drug class being prescribed. Both patient and clinician characteristics have been examined as important contributors to the decision to prescribe. For example, clinicians’ experience of time pressure may have influenced opioid prescribing, where even within an individual physician’s schedule, the likelihood that an appointment resulted in an opioid prescription increased in primary care outpatient setting as the workday progressed and as appointments ran behind schedule [1]. Intensity of benzodiazepine prescribing was less likely in female clinicians [2]. In nursing home settings, higher clinician-level prescribing intensities of antibiotics are correlated with higher prescribing intensities of benzodiazepines and of opioids, while a clinician’s prescribing propensity of antibiotics is an important determinant of initiating benzodiazepines or opioids, even accounting for nursing home resident characteristics [2–3]. Decision to prescribe and associated differences in prescribing intensity may have long-lasting impacts on patients. For example, one study in emergency departments showed that patients treated by high-intensity opioid prescribers developed higher rates of long-term opioid use [4].

Identifying and understanding the sources of variation or systematic differences in prescribing intensities may help improve patient safety and outcomes. Tjia, Gurwitz, and Briesacher characterized “prescribing cultures” in long-term care settings, and such cultures likely operate in other health care delivery settings [5]. When considering prescribing culture, the number of clinicians per facility could represent availability of patient care resources. Larger facilities may have more resources to support appropriate prescribing—e.g., clinical pharmacists for consultation or availability of nonpharmacological treatments like psychotherapy—relative to smaller facilities, which could influence facility-level medication intensity and associated patient outcomes and quality of care. Qualitative studies of prescribing have not directly linked organization size with clinician prescribing [6–9], though a number of related factors have been noted as important, including limited alternative treatment options [6], access to resources [7], and lack of staff and time [9].

The Veterans Health Administration (VHA) is the largest integrated health care system in the U.S., with 140 medical centers of varying sizes across the country. In this analysis, we examined whether the number of clinicians in each VHA facility (henceforth, “facility clinicians”) has an association with facility prescribing intensity in ambulatory settings. We focused on several commonly prescribed medication classes including benzodiazepines, antipsychotics, antiepileptics, antidepressants, antibiotics, and opioids. We chose the set of medications following a similar approach to Quinn et al. [3], examining antibiotics, which are used for episodic treatment, and psychotropic and opioid medications, prescribing of which is more long-term and potentially sensitive to other treatment resources available locally. We first examined the relationship between intensity and number of clinicians in 2017. We then examined 2014 to 2017 data to consider if the relationship was stable over time.

## Methods

We used the VHA Corporate Data Warehouse, the central data repository derived from the VA’s systemwide electronic health record, to calculate facility prescribing intensity for each medication, separately for primary care physicians (PCPs) and for psychiatrists. For psychiatrists, we calculated intensity measures for benzodiazepines, antipsychotics, antiepileptics, and antidepressants; for PCPs, we examined antibiotics, opioids, antiepileptics, and antidepressants. To calculate facility medication prescribing intensity, we first identified patient-clinician dyads, classified clinicians as a primary care physician (PCP) or a psychiatrist, identified VHA facility of each clinician, calculated clinician-level medication prescribing intensities for each medication, and calculated facility-level medication prescribing intensity by averaging across clinician-level medication prescribing intensities of each medication class separately for PCPs and for psychiatrists.

Specifically, we identified a cohort of clinicians who had at least one outpatient or inpatient encounter with Veterans who were 55 years or older and alive on January 1 of 2017 and created a dataset containing unique patient-clinician dyads based on all encounters in 2017. We limited our analyses to Veterans 55 years or older because this is a secondary analysis of a parent project focused on prescribing among older Veterans [10]. We included patient-clinician dyads at the encounter level; that is, if a patient had two encounters with two different clinicians in 2017, we created two separate patient-clinician dyad records that included the same patient (e.g., Clinician A and Patient X; Clinician B and Patient X). We classified all physicians as a PCP, psychiatrist, or other (not used for this study) using National Clinician Identifiers (NPIs) and the National Plan and Provider Enumeration System (NPPES) dataset. We excluded physicians without an NPI or missing taxonomy in NPPES as well as those with a primary taxonomy of student and without a secondary taxonomy. We assumed those with a primary taxonomy of student and a non-missing secondary taxonomy represented resident physicians, which we excluded in the sensitivity analysis (see **Statistical Analysis**). We assigned each clinician to a single VHA facility based on the plurality of encounters in 2017, assigning the clinician to the facility of their last encounter in 2017 in the case of a tie.

We identified all outpatient prescription fills among the included Veterans for the six medication classes of interest (see Supplementary Table 1). Based on the patient-clinician dyad dataset, we computed two measures of clinician-level prescribing intensity for each medication class of interest: (1) percentage of each physician’s patients prescribed at least one medication out of all patients that a given physician had any encounters and (2) mean number of days supply of the medication per patient for each clinician. We then generated facility-specific clinician prescribing intensity measures for the medication classes of interest by taking the average of each clinician-level medication prescribing intensity measures separately for each specialty. After examining intensity in 2017, we repeated our analysis using additional years of 2014 to 2016. For each facility, we also obtained the number of psychiatrists and PCPs used in the calculation of prescribing intensity.

### Statistical analysis

We conducted analyses separately for each medication class and clinician type. For each facility, we summarized the number of clinicians included in the prescribing intensity measure and the two clinician-level prescribing intensity measures (i.e., percentage of patients prescribed at least one medication and mean number of days supply per patient). To examine the relationship between facility medication prescribing intensity and the number of facility clinicians, we used scatter plots and a locally estimated scatterplot smoothing (loess) line. For all classes, the visualizations consistently showed a non-linear near L-shaped relationship across clinician type and medication class, which guided the modeling strategy. We modeled prescribing intensity measures using a generalized linear mixed-model with facility number of clinicians as the primary predictor and log link to reflect the L-shaped non-linearly decreasing prescribing intensities with increasing number of clinicians.

Based on the model, we summarized the relationship by examining the changing rate (slope) of prescribing intensity associated with an increase of 10 prescribing clinicians. To assess if the patterns observed in 2017 data reflected consistent associations across years, we visualized and modeled the relationships each year from 2014 to 2016. Because the associations were consistent across years, we combined the data across the four years and fit a generalized linear mixed model with facilities as random intercepts to account for potential within-facility correlation. We included indicators for year (reference = 2014) and an interaction between number of facility clinicians and year to allow differential rates of change in prescribing intensity by year. For models that did not detect a statistically significant interaction (based on a 3 degrees of freedom likelihood ratio test with a significance level of 0.05), we refit the model without the interaction terms and estimated an overall rate averaged across years, adjusting for different yearly levels of prescribing intensity.

We conducted three sensitivity analyses. First, we repeated analyses without excluding clinicians with small panels, i.e., without excluding clinicians with fewer than 10 patients. Second, we repeated analyses after excluding resident physicians. Third, we assessed if the relationship remained after further adjusting for facility number of beds and patient volume, which indirectly assess facility patient care capacity. Statistical analysis was performed using Stata 17.0 (College Station, TX). This study used de-identified patient data, and the parent study [10] was approved by the VA Ann Arbor institutional review board (IRB) approval.

## Results

We included data from 140 VHA facilities in 2017. We initially identified 13,347,658 unique patient-clinician dyads and excluded 3.6% of the dyads because the encounter clinician lacked an NPI or included a medical student. In 2017, the mean number of facility psychiatrists with at least 10 patients was 29.9 (SD = 22.1, range = 3, 111) and the mean number of facility PCPs with at least 10 patients was 114.9 (SD = 77.7, range = 13, 367).

**Fig. 1:**
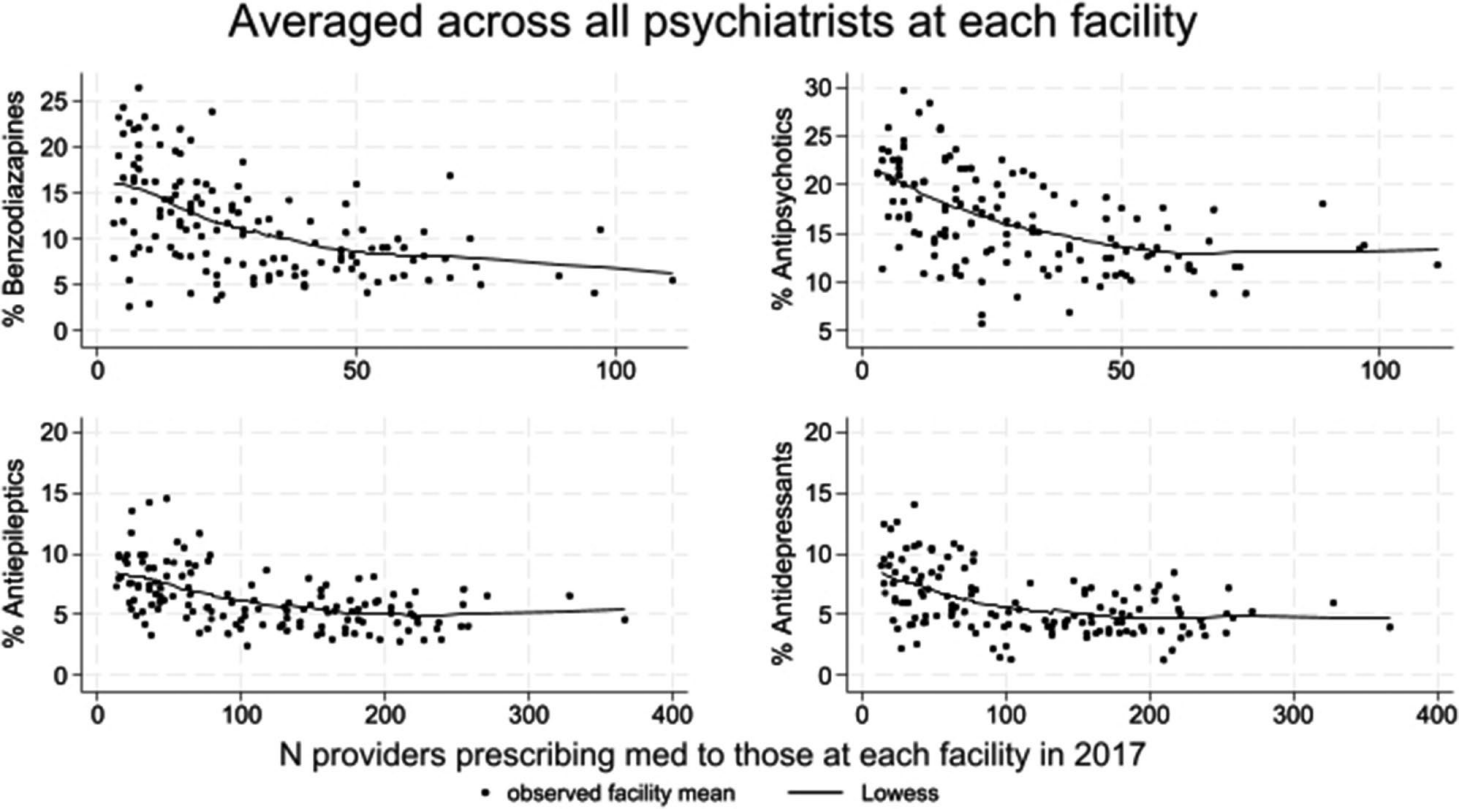
Relationship between clinician-level percentage of patients on each of benzodiazepine, antipsychotics, antiepileptics and antidepressants averaged across all facility psychiatrists versus number of facility psychiatrists in 2017

**Fig. 2:**
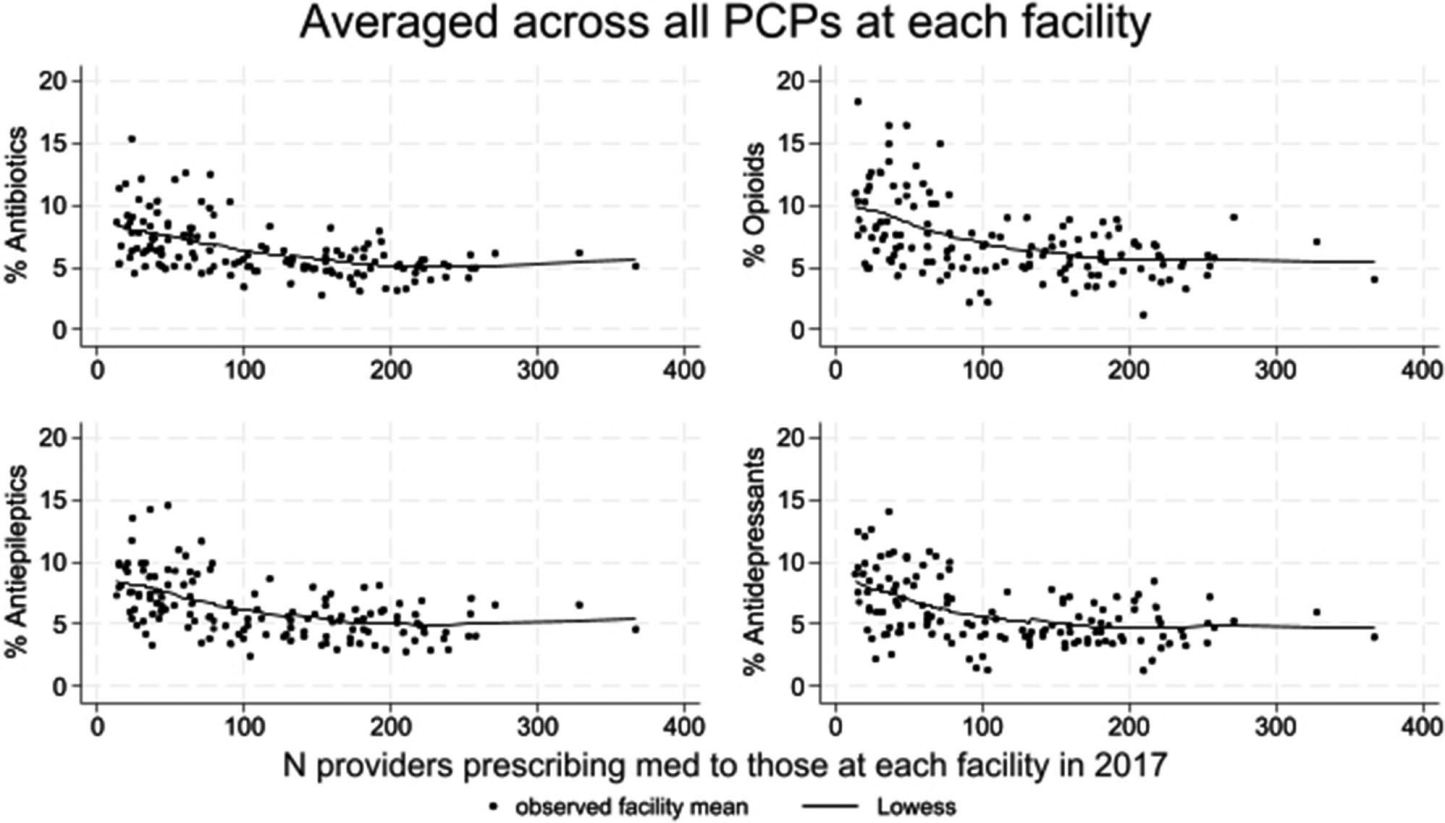
Relationship between clinician-level percentage of patients on each of antibiotics, opioids, antiepileptics and antidepressants averaged across all primary care physicians (PCPs) versus number of facility PCPs in 2017

Figure 1 shows the relationship between clinician-level percentage of patients who filled prescriptions for benzodiazepines, antipsychotics, antiepileptics and antidepressants averaged across each facility’s prescribing psychiatrists versus the facility-level number of psychiatrists in 2017. Figure 2 shows the relationship between clinician-level percentage of patients on each of antibiotics, opioids, antiepileptics and antidepressants averaged across facility’s prescribing PCPs versus the facility-level number of PCPs in 2017. For both psychiatrists and PCPs, facility prescribing intensities of each medication class examined decreased as the number of facility clinicians increased. Although not shown, for both psychiatrists and PCPs, mean numbers of days supply also showed the same pattern of decreasing facility prescribing intensity with increasing number of facility clinicians for all classes examined.

Table 1 summarizes the facility prescribing intensity measures for each medication (% of patients prescribed and mean number of days supply per patient) and the rate of change in the facility prescribing intensity associated with additional facility clinicians. All rate estimates demonstrated a significant decline in prescribing intensity with increasing number of facility clinicians. For example, for psychiatrists, the benzodiazepine rate of 0.89 (CI = 0.86, 0.92) means that for a facility with 10 additional psychiatrists, that facility’s psychiatrists, on average, had 11% fewer patients on their respective panels prescribed a benzodiazepine. Across the classes examined for each specialty, psychiatrists had a larger magnitude of rate of decline than PCPs, ranging from 0.89 [benzodiazepines] to 0.94 [antiepileptics] versus 0.97 [opioids and antiepileptics] to 0.98 [antibiotics and antidepressants].

**Table 1:** Clinician-level^*^ prescribing intensity for psychiatrists and primary care clinicians across all VA facilities and rate of change associated with 10 additional facility clinicians, 2017

	Percent of Patients on Each Medication							
**By Psychiatrists**	Benzodiazepines		Antipsychotics		Antiepileptics		Antidepressants	
%, mean (SD)	11.5	(5.4)	16.4	(4.9)	10.7	(3.8)	48.1	(13.4)
Rate^†^ (95% CI)	0.89	(0.86, 0.92)	0.93	(0.90, 0.95)	0.94	(0.92, 0.96)	0.93	(0.91, 0.95)
**By PCPs**	Antibiotics		Opioids		Antiepileptics		Antidepressants	
%, mean (SD)	6.4	(2.2)	7.1	(3.1)	6.3	(2.4)	5.9	(2.5)
Rate^†^ (95% CI)	0.98	(0.97, 0.98)	0.97	(0.97, 0.98)	0.97	(0.96, 0.98)	0.98	(0.97, 0.98)
	**Mean Days Supply per Patient**							
**By Psychiatrists**	Benzodiazepines		Antipsychotics		Antiepileptics		Antidepressants	
Days, mean (SD)	21.1	(11.0)	33.5	(12.9)	18.6	(8.4)	132.8	(48.9)
Rate^†^ (95% CI)	0.89	(0.86, 0.92)	0.91	(0.88, 0.93)	0.91	(0.88, 0.94)	0.92	(0.90, 0.94)
**By PCPs**	Antibiotics		Opioids		Antiepileptics		Antidepressants	
Days, mean (SD)	1.5	(0.5)	9.1	(5.0)	11.5	(4.9)	11.7	(6.0)
Rate^†^ (95% CI)	0.97	(0.97, 0.98)	0.97	(0.96, 0.98)	0.97	(0.96, 0.98)	0.97	(0.96, 0.98)

Abbreviations: SD is standard deviation; CI is confidence interval; PCP is primary care physician

^*^Clinicians include residents or attendings with at least 11 patients in their patient panel in 2017

^†^Estimated by exponentiating 10 times the coefficient of the number of facility clinicians from a multilevel generalized mixed model of prescribing intensity with log link and facilities as random intercepts. If the model did not fit, generalized linear model with robust standard deviation was used to account for within facility correlation

To examine intensity in additional years of data, we first established that the mean number of facility clinicians remained stable from 2014 to 2017 (Supplementary Table 2). We also saw that averaged across facilities, the percentage of patients on each medication declined over the years for antipsychotics and benzodiazepines in psychiatrists and for opioids in PCPs. For example, the percentage of patients prescribed benzodiazepine fell from 17.0% (2014) to 11.5% (2017) among psychiatrists. In contrast, the percentage for antiepileptics rose in both clinician types: from 10.3 to 10.7% among psychiatrists and from 5.5 to 6.3% among PCPs. Across years, minimal change occurred in antibiotic prescribing by PCPs or antidepressants by both psychiatrists and PCPs. Facility averages of mean number of days supply for different medication classes demonstrated similar trends across all years (not shown).

Table 2 shows the rate of change in the two prescribing intensity measures associated with 10 additional facility clinicians by each study year. As with our analysis of 2017, for all medications across the additional years of 2014 to 2016, prescribing intensity decreased significantly with more facility clinicians. For most medication classes, we did not find a significant interaction for number of facility clinicians by year and thus reported a summary rate across years 2014 to 2017. For example, the estimated rate of decline for psychiatrists in the percentage of their patients prescribed a benzodiazepine was 0.89, 0.89, 0.89 and 0.90 for each of the years from 2014 to 2017, respectively, with an overall rate across the four years of 0.89 (*p* < 0.001). Our findings of significantly decreasing prescribing intensity with increasing number of clinicians remained after adjusting for bed size and patient volume. Lastly, we also found similar results when including data from clinicians with less than 10 patients as well as after excluding residents.

**Table 2:** Rates* of facility-level decline in prescribing intensity for psychiatrists and primary care clinicians associated with VA facility size, 2014 to 2017

	Psychiatrists								
	Percentage of patients prescribed class					Mean days supply			
	BZD	AP	AE	AD		BZD	AP	AE	AD
2014	0.89	0.94	0.93	0.94		0.85	0.88	0.88	0.89
2015	0.89	0.94	0.94	0.94		0.86	0.89	0.89	0.90
2016	0.89	0.93	0.94	0.93		0.84	0.87	0.88	0.89
2017^†^	0.90	0.94	0.95	0.94		0.87	0.89	0.90	0.90
p-value^‡^	0.40	0.23	0.33	0.22		0.03	0.03	0.06	0.08
Overall^§^	0.89	0.94	0.94	0.94		--	--	0.89	0.89
	Primary Care Physicians								
	Percentage of patients prescribed class					Mean days supply			
	AB	Opioids	AE	AD		AB	Opioids	AE	AD
2014	0.98	0.97	0.97	0.97		0.98	0.96	0.96	0.97
2015	0.98	0.97	0.97	0.97		0.98	0.96	0.96	0.97
2016	0.98	0.97	0.97	0.97		0.98	0.96	0.96	0.97
2017^†^	0.98	0.97	0.97	0.97		0.98	0.96	0.96	0.96
p-value^‡^	0.28	0.76	0.56	0.24		0.81	0.84	0.45	0.26
Overall^§^	0.98	0.97	0.97	0.97		0.98	0.96	0.96	0.96

Abbreviations: BZD is benzodiazepines; AP is antipsychotics; AE is antiepileptics; AD is antidepressants

*Rates estimated the change in prescribing intensity outcome associated with 10 additional facility clinicians, based on prescribing intensity data from 2014 to 2017 modeled using multilevel generalized mixed models with log link and facilities as random intercepts and number of facility clinicians in each year, year indicators, and their interaction terms as predictors. All rate estimates were significant (*p* < 0.001) when tested for rates equal to 1.0

^†^ 2017 estimates may slightly differ from those in Table 1 as these are based on a model using data from all years

^‡^ P-value is from the 3-degrees of freedom likelihood ratio test (LRT) for slope differences across years, i.e., from testing for the interaction terms of number of providers by year indicators

^§^ An overall rate (i.e., slope across the four years) is estimated based on a mixed model with each prescribing intensity measure after dropping the interaction terms if the LRT test described in footnote b is not significant (*p* > 0.05). An overall rate is not estimated when the LRT of differential slope by year was significant

## Discussion

Our study showed a lower prescribing intensity among psychiatrists and PCPs as the number of clinicians per facility increased. This finding remained consistent across two specialties, multiple medication classes, and multiple years of analyses. Psychiatrists had a larger decrease in prescribing intensity at facilities with more clinicians than among PCPs, which potentially reflects higher prescribing rates among medication classes examined for psychiatrists (benzodiazepines, antipsychotics, antiepileptics, and antidepressants) than those examined for PCPs (antibiotics, opioids, antiepileptics, and antidepressants).

The association of prescribing intensity with facility size remaining consistent across the years examined (2014 to 2017) suggests stability within the VHA. On the other hand, the differences in prescribing intensity across facilities within the VHA system are striking given ongoing quality improvement initiatives [11,12]. However, we did observe overall declining prescribing intensity over the study years for medications with black box warning or safety initiatives, including antipsychotics among psychiatrists and opioids among PCPs, consistent with what others have shown [10–13]. This suggests that despite national improvements in prescribing appropriateness, individual facilities may benefit from more concentrated resources. On the other hand, antiepileptic prescribing—most notably gabapentin—increased over this same period, also consistent with previous reports [11,14]. Notably, the association of reduced prescribing intensities with increasing number of facility clinicians seen across multiple medication classes remained significant even in these cases where use of these specific classes increased (e.g., antiepileptics) or decreased (e.g., benzodiazepine) over the study years.

Possible explanatory factors for variation in prescribing intensities include patient, clinician, organizational, and environmental factors. To our knowledge, literature has not previously characterized the association of prescribing intensity with the number of facility clinicians. Facilities with more clinicians may allow for a greater degree of specialization, which may in turn lead to more judicious pharmacotherapy. In addition, clinicians in larger facilities may have more clinical resources to support optimal care delivery, including specialty referral. Although number of clinicians, bed size, or patient volume do not necessarily directly reflect the breadth of facility clinical capacity, smaller facilities have fewer resources to support specialty care or alternative treatment options, including psychotherapy and specialty mental health care clinics. Of note, we found the declining rate of prescribing intensity with increasing number of facility clinicians to hold even after adjusting for facility bed size and patient volume. Ultimately, these findings add to the evidence that multiple factors influence clinical decision-making related to prescribing decisions unrelated to patient clinical characteristics [1,2].

This study includes several limitations. The study included Veterans 55 years or older; although we have no reason to believe this to be the case, study findings may have differed among younger patients. In addition, our analysis did not account for potentially different patient characteristics between facilities. However, higher prescribing in facilities with smaller numbers of clinicians is likely not because smaller facilities tend to treat patients for whom these psychotherapies are more appropriate. We classified physicians using NPIs and NPPES data, which may not reflect the most up-to-date physician specialties; however, we do not anticipate that this influenced our findings. Finally, we cannot determine whether our findings generalize outside the VHA as majority Veteran patient population are males. Additionally, across the VA medical centers, psychiatrists are integrated with primary care clinics and Veterans in primary care have direct access to mental health resources and support teams which may represent VA specific prescribing environment and culture.

Given the consistency in our findings across medication classes and years, others may seek to replicate and extend the findings with more recent data and in other patient care settings, including nursing homes, hospices, or emergency departments, as well as replicated within community settings. Considering factors associated with prescribing intensity can inform approaches to support improved patient care. Future work should explore mechanisms underlying relationships between prescribing intensity and the number of facility clinicians to promote increased use of appropriate but underutilized medications (e.g., pharmacotherapy for alcohol use disorder) or reduce use of overprescribed medications (e.g., anticholinergic medications to older adults).

### Electronic supplementary material

Below is the link to the electronic supplementary material.

**Supplementary Material 1**: Supplementary Table 1. Medications contributing to each medication class. Supplementary Table 2. Number of clinicians and clinician-level percentage of patients on each medication class, averaged across facilities; each clinician had to have at least 11 patients in their patient panel and residents are included as clinicians

## Data Availability

Data are not available.
