# Supplementary material for: Relationship between facility number of clinicians and prescribing intensity of psychotropic medications, opioids, and antibiotics in ambulatory practice
Source: BMC Health Serv Res. 2024 Feb 16;24:217. doi: 10.1186/s12913-024-10613-z (PMC10874022; doi:10.1186/s12913-024-10613-z)

**Supplementary Table 1.** Medications contributing to each medication class

| **Medication Class** | **AHFS Class(es)** | **Medications Contributing** |
| --- | --- | --- |
| Benzodiazepines | 28:24.08 | Alprazolam Chlordiazepoxide Clorazepate Diazepam Estazolam Flurazepam Lorazepam Midazolam Oxazepam Quazepam Temazepam Triazolam Clobazam Clonazepam Prazepam Halazepam |
| Opioids | 28:08.08 and  28:08.12 | Codeine Fentanyl Hydrocodone Hydromorphone Levorphanol Meperidine Methadone Morphine Opium Oxycodone Oxymorphone Remifentanil Sufentanil Tapentadol Tramadol Buprenorphine Butorphanol Nalbuphine Pentazocine Dihydrocodeine |
| Antidepressants | 28:16.04.XX | Isocarboxazid Phenelzine Tranylcypromine Rasagiline Selegiline Desvenlafaxine Duloxetine Levomilnacipran Venlafaxine Milnacipran Citalopram Escitalopram Fluoxetine Fluvoxamine Paroxetine Sertraline Nefazodone Trazodone Vilazodone Vortioxetine Amitriptyline Amoxapine Clomipramine Desipramine Doxepin Imipramine Maprotiline Nortriptyline Protriptyline Trimipramine Bupropion Mirtazapine |
| Z-drugs | Subset of  28:24.92 | Eszopiclone Zaleplon Zolpidem |
| Antiepileptics | 28.12.XX.XX  except  benzodiazepines | Ethotoin Fosphenytoin Phenytoin Ethadione Paramethadione Trimethadione Ethosuximide Methsuximide Brivaracetam Carbamazepine Eslicarbazepine Felbamate Gabapentin Lacosamide Lamotrigine Levetiracetam Magnesium Sulfate Oxcarbazepine Perampanel Pregabalin Rufinamide Sultiame Tiagabine Topiramate Valproate Divalproex Valproic Acid Vigabatrin Zonisamide Acetazolamide Primidone Methohexital |
| Antipsychotics | 28.16.08.XX | Aripiprazole Asenapine Brexpiprazole Cariprazine Clozapine Iloperidone Lurasidone Olanzapine Paliperidone Pimavanserin Quetiapine Risperidone Ziprasidone Haloperidol Chlorpromazine Fluphenazine Perphenazine Prochlorperazine Thioridazine Trifluoperazine Thiothixene Loxapine Molindone Pimozide |
| Antibiotics | 8:12 | Multiword single medications:   - Imipenem and Cilastatin - Ceftolozane Sulfate and Tazobactam Sodium - Penicillin G Benzathine - Penicillin G Potassium, Penicillin G Sodium - Penicillin G Procaine - Penicillin V - Piperacillin and Tazobactam - Ticarcillin and Clavulanate - Polymyxin B - Quinupristin/Dalfopristin - Fusidate Sodium   Single word medications:  Amikacin Gentamicin Neomycin Streptomycin Tobramycin Paromomycin  Cefadroxil Cefazolin Cephalexin  Cefaclor Cefprozil Cefuroxime cefotetan cefoxitin  Cefdinir Cefditoren Cefixime Cefotaxime Cefpodoxime ceftazidime Ceftibuten ceftriaxone  Cefepime Ceftaroline Ceftobiprole  Doripenem Ertapenem Meropenem  cefotetan cefoxitin Aztreonam Chloramphenicol Erythromycin Telithromycin Azithromycin Clarithromycin Fidaxomicin Amoxicillin Ampicillin Dicloxacillin Nafcillin Oxacillin Ciprofloxacin Delafloxacin Gemifloxacin levofloxacin Moxifloxacin Ofloxacin Co-trimoxazole sulfadiazine sulfasalazine Demeclocycline Doxycycline Minocycline Tetracycline Tigecycline Bacitracin Daptomycin Dalbavancin Oritavancin Teicoplanin Telavancin Vancomycin Clindamycin Lincomycin Linezolid Tedizolid Colistimethate rifaximin Rifabutin rifampin Rifapentine |

**Supplementary Table 2**: Number of clinicians and clinician-level percentage of patients on each medication class, averaged across facilities; each clinician had to have at least 11 patients in their patient panel and residents are included as clinicians.

|  | Clinicians | | BZD | | | AP | | | AD | | Antiepileptics | |
| --- | --- | --- | --- | --- | --- | --- | --- | --- | --- | --- | --- | --- |
|  | (N) | | (%) | | | (%) | | | (%) | | (%) | |
| **Psychiatrists** | Mean | SD | Mean | SD | mean | | SD | mean | | SD | mean | SD |
| 2014 | 29.4 | 21.7 | 17.0 | 8.3 | 17.3 | | 5.3 | 48.8 | | 13.0 | 10.3 | 4.0 |
| 2015 | 29.7 | 22.0 | 15.2 | 7.5 | 16.9 | | 5.0 | 48.9 | | 13.3 | 10.4 | 3.7 |
| 2016 | 30.0 | 22.3 | 13.7 | 6.7 | 16.9 | | 5.3 | 49.3 | | 13.8 | 10.6 | 3.9 |
| 2017 | 29.9 | 22.1 | 11.5 | 5.4 | 16.4 | | 4.9 | 48.1 | | 13.4 | 10.7 | 3.9 |
|  |  |  |  |  |  | |  |  | |  |  | |
|  | Clinician | | Antibiotics | | | Opioids | | | AD | | Antiepileptics | |
|  | (N) | | (%) | | | (%) | | | (%) | | (%) | |
| **PCP** | Mean | SD | Mean | SD | mean | | SD | Mean | | SD | Mean | SD |
| 2014 | 111.4 | 77.2 | 6.5 | 2.1 | 10.0 | | 4.2 | 5.7 | | 2.3 | 5.5 | 2.1 |
| 2015 | 114.4 | 77.8 | 6.5 | 2.2 | 9.3 | | 4.0 | 5.7 | | 2.3 | 5.7 | 2.2 |
| 2016 | 114.3 | 78.5 | 6.3 | 2.1 | 8.3 | | 3.5 | 5.8 | | 2.4 | 6.1 | 2.3 |
| 2017 | 114.9 | 77.7 | 6.4 | 2.2 | 7.1 | | 3.1 | 5.9 | | 2.5 | 6.3 | 2.4 |

Abbreviations: PCP is primary care physicians; BZD is benzodiazepines; AP is antipsychotics; AD is antidepressants


Supplement: Supplementary file 1 — Supplementary Material 1: Supplementary Table 1. Medications contributing to each medication class. Supplementary Table 2. Number of clinicians and clinician-level percentage of patients on each medication class, averaged across facilities; each clinician had to have at least 11 patients in their patient panel and residents are included as clinicians [file 12913_2024_10613_MOESM1_ESM.docx]
